# THE INFLUENCE OF GLOBAL CLIMATE CHANGE ON THE SCIENTIFIC FOUNDATIONS AND APPLICATIONS OF ENVIRONMENTAL TOXICOLOGY AND CHEMISTRY: INTRODUCTION TO A SETAC INTERNATIONAL WORKSHOP

**DOI:** 10.1002/etc.2037

**Published:** 2012-12-18

**Authors:** Ralph G Stahl, Michael J Hooper, John M Balbus, William Clements, Alyce Fritz, Todd Gouin, Roger Helm, Christopher Hickey, Wayne Landis, S Jannicke Moe

**Affiliations:** † DuPontWilmington, Delaware, USA; ‡ U.S. Geological SurveyColumbia, Missouri, USA; § National Institutes of HealthBethesda, Maryland, USA; ‖ Colorado State UniversityFort Collins, Colorado, USA; # National Oceanic and Atmospheric AdministrationSeattle, Washington, USA; †† UnileverSharnbrook, United Kingdom; ‡‡ U.S. Fish & Wildlife ServiceArlington, Virginia, USA; §§ National Institute of Water and Atmospheric ResearchHamilton, New Zealand; ‖‖ Western Washington UniversityBellingham, Washington, USA; ## Norwegian Institute for Water ResearchOslo, Norway

**Keywords:** Global climate change, Chemical contaminant, SETAC Workshop, Risk assessment, Interaction

## Abstract

This is the first of seven papers resulting from a Society of Environmental Toxicology and Chemistry (SETAC) international workshop titled “The Influence of Global Climate Change on the Scientific Foundations and Applications of Environmental Toxicology and Chemistry.” The workshop involved 36 scientists from 11 countries and was designed to answer the following question: How will global climate change influence the environmental impacts of chemicals and other stressors and the way we assess and manage them in the environment? While more detail is found in the complete series of articles, some key consensus points are as follows: (1) human actions (including mitigation of and adaptation to impacts of global climate change [GCC]) may have as much influence on the fate and distribution of chemical contaminants as does GCC, and modeled predictions should be interpreted cautiously; (2) climate change can affect the toxicity of chemicals, but chemicals can also affect how organisms acclimate to climate change; (3) effects of GCC may be slow, variable, and difficult to detect, though some populations and communities of high vulnerability may exhibit responses sooner and more dramatically than others; (4) future approaches to human and ecological risk assessments will need to incorporate multiple stressors and cumulative risks considering the wide spectrum of potential impacts stemming from GCC; and (5) baseline/reference conditions for estimating resource injury and restoration/rehabilitation will continually shift due to GCC and represent significant challenges to practitioners. Environ. Toxicol. Chem. 2013;32:13–19. © 2012 SETAC

## INTRODUCTION

Global climate change (GCC) is a powerful advancing force 1, 2 that can impact ecosystems and humans for decades to come 3–6. It is likely that GCC will manifest as a suite of stressors, or a syndrome, impacting the abiotic, biotic, and socioeconomic 3 components of the landscape 7. It is possible that changes in stressor regimes 8 and the potential for toxic effects will occur, as well as a need to revise assumptions about past conditions being models for current and future conditions 9, 10. In addition, changes in current methods and a reexamination of predictions and predictive tools for the fate and transport of chemical stressors 11 that are built on those assumptions 12, 13 will be needed. These points are particularly important as the chemical fate and dynamics data are critical for the performance of human health risk assessment and ecological risk assessments 7, 9, 14 as well as the practice of damage assessment and restoration of natural resources in the United States 15, 16 and Europe 3, 17, 18.

Recently, Wenning et al. 19 argued that environmental toxicologists and chemists, who have tended to be absent from much of the international debate on GCC and its effects, need to keep pace with these changing conditions 20 and join the debate. To address this point, a Society of Environmental Toxicology and Chemistry (SETAC) workshop was held to explore the potential influence of GCC on the foundation and applications of environmental toxicology and chemistry. Some 36 scientists, managers, policy makers, and students from 11 countries participated and answered two main questions: (1) What are the potential impacts of GCC on the scientific foundations of environmental toxicology and chemistry? (2) How might those impacts influence the application of the science to chemical risk and natural resource injury and recovery assessments?

This is the first of seven papers from this workshop and provides a short survey of the relevant literature, a description of the workshop's organizational basis, and a summary of overarching consensus items. Detailed results from the workshop can be found in the individual papers 21–26.

## FRAMEWORK FOR ADDRESSING THE ISSUE

To address the potential for changes in the practice of environmental toxicology and chemistry wrought on various scientific and regulatory levels by GCC, we organized the workshop around two major technical categories—foundations and applications. To focus our discussions further, the foundations category was divided into three major subcategories that provide data for regulatory assessments: chemical fate and modeling, toxicological mechanisms, and populations and communities. For this workshop, these three subcategories represented the technical inputs that are integrated into applications such as risk- and injury-assessment paradigms 27, 28 and ultimately used to inform risk-management decisions. These same inputs are used not only in assessing injury to natural resources but also to help determine what actions may be needed to restore those harmed resources 29, 30. Similarly, managers of river basins in the European Union are required to consider both the sensitivity of aquatic ecosystems and the resilience of restoration measures to potential GCC impacts in their management plans for the future 31. Restoration and rehabilitation efforts in Australia are based on similar technical inputs and frequently include an assessment of potential impacts from GCC 32, 33. Other subcategories of inputs to these types of assessments are possible, but the three foundations subcategories were considered most important to considering the potential influence of GCC on environmental toxicology and chemistry.

The application category included the conduct of human health and ecological risk assessments and the assessment of injuries to natural resources. Exploration of the influence of a changing environment on human health risk assessment, ecological risk assessment, and assessment of harm to natural resources required placing GCC in context, based on what is known to date from the current literature. For the purposes of this workshop, we viewed direct effects of GCC mostly as abiotic, including increased temperature 34 and modification of hydrological cycles, which then may have repercussions for local weather and runoff events 35. In addition, there may be changes in soil characteristics 36, the intensity of fire and its frequency of occurrence, changes in the temperature and chemistry profiles of oceanic areas, in addition to rising sea levels 37. As Solomon et al. 6 have detailed, additional direct impacts from GCC can be found on glacial and polar ice, snow pack, and duration that illustrate a general lack of stationarity in the variability of these physical and chemical endpoints.

Indirect effects of GCC arise from modifications of ecological structures or processes by direct effects of GCC and can be demonstrated as combinations of direct and indirect effects such as desynchronization of forage/forager or predator/prey phenologies 38, elimination or displacement of historical ranges and assemblages of species 16, influx of invasive species or diseases that benefit from changing climate conditions 39, and formation of novel communities and ecosystems with new interspecies interactions 5. Moreover, indirect GCC impacts can arise from changes in human activities in response to GCC. Humans, for example, can change energy development and agricultural practices through mitigation and adaptation effort 40 or move themselves in some cases to geographies less impacted by GCC. These latter points illustrate the need to understand the potential influence of GCC not only on the foundations of environmental toxicology and chemistry but also on the applications of these foundations in the assessment of chemicals in the environment.

## SUMMARY OF RESULTS

A summary of key findings from the individual papers resulting from the workshop is provided in Table 1 and discussed, along with a brief synopsis of the relevant literature, in the following sections.

**Table 1:** Key consensus points related to the influence of global climate change (GCC) on the foundations and application of environmental toxicology and chemistry; additional details on each topic area can be found in the cited references

Topic area	Summary of consensus points	Reference
Occurrence of chemicals	• Quantifying the influence of GCC on bioavailability of chemical contaminants represents an area of ongoing research.	21
	• It is critically important that high-quality monitoring networks in all regions of the world are established and maintained to improve our assessment of “baseline” conditions. The utility of the monitoring networks should be aimed at improving our overall understanding of processes that influence variability of data.	
	• Output of environmental modeling implies that changes in human activity resulting in decreased or increased emissions to the environment of chemicals will have a more significant influence on exposure, as opposed to the effects of climate on the transport and fate of chemicals.	
	• Uncertainty in input parameters for physical/chemical properties for use in models tends to be greater than uncertainty due to changes in the environment brought about by GCC.	
	• Caution is needed when interpreting and/or speculating on the importance of GCC with respect to how changes due to GCC will influence the fate and bioavailability of chemicals.	
Mechanisms of toxicity	• Mechanistic data, including new approaches in biological and computational sciences, can facilitate understanding how the effects of toxicants will interact with direct and indirect effects of GCC.	22
	• Adverse outcome pathways (AOPs) allow for identification of knowledge gaps and translation of complex mechanistic data on the interaction of toxicants and GCC into an outcome relevant to risk and damage assessments.	
	• GCC can affect the mechanisms for both GCC and toxicant adaptation/acclimation. For example, toxicants can influence the sensitivity of organisms to climate, and climate can influence the sensitivity of organisms to toxicants.	
	• The AOP approach can be applied to understanding the interactions of GCC with chemical toxicants. This can be done prospectively to predict potential effects in natural and susceptible populations/regions or retrospectively to discern mechanisms of damage to a particular ecosystem, population, or individual. It may also be used to predict effects in other systems subject to similar conditions resulting from or influenced by GCC.	
Populations and communities	• Combined effects of contaminants and GCC are mediated by ecological and evolutionary processes at different spatial and temporal scales.	23
	• Indirect impacts of GCC may be more important than direct impacts.	
	• Impacts from GCC may be slow and therefore difficult to distinguish from natural variation.	
	• Ecological responses to environmental stressors are often nonlinear, and GCC may increase the risk of ecological systems exceeding thresholds/tipping points and reaching alternative stable states.	
	• GCC may favor opportunistic species with high potential for reproduction and dispersal and may therefore benefit pest species.	
	• Species and communities identified as vulnerable to GCC (e.g., amphibians, coral reefs, polar species) are likely to be particularly vulnerable to interactions between GCC and other stressors.	
Human health risk assessment	• Small changes in exposure variability and/or vulnerability to chemicals or other toxicants can lead to large changes in risk and large uncertainties.	24
	• GCC is likely to lead to increases in variability and bidirectional changes in exposure of humans to chemicals and other toxicants This may result from changes in human use patterns of pesticides as well as from changes in the fate and transport of those substances stemming from GCC.	
	• Monitoring and sampling of exposures of humans to chemicals and other toxicants should be done with frequency sufficient to capture altered variability that may result from GCC.	
	• Increased vigilance and action will be needed to lessen potential gaps in policies or regulatory actions to protect people from unacceptable exposure to chemicals and other toxicants in both developed and developing countries.	
Ecological risk assessment	• Future ecological risk assessments will need to use a multistressor approach to reflect potential influences of GCC, including chemical and nonchemical stressors relevant to GCC.	25
	• Ecosystem services can and should be applied in the ecological risk-assessment process as assessment end points.	
	• In the future, ecological risk assessments will need to consider management scenarios in the problem-formulation step, particularly as GCC impacts become manifest.	
	• Systems will likely change to unprecedented extents and at unpredictable rates, meaning that monitoring, adaptive management, and ongoing ecosystem studies are essential to manage for high uncertainty.	
	• Consideration for Type III error (asking the wrong question to begin with) will be important to include in ecological risk assessments.	
Damage to natural resources, their restoration/rehabilitation	• Shifting and increased the variability of baseline and/or reference condition will present increased challenges for damage assessment, restoration, and/or rehabilitation planning and implementation.	26
	• Incorporating insights from cumulative risk assessments will enhance the likelihood of successful restoration and/or rehabilitation efforts.	
	• Assessments of vulnerability of important organisms and habitats to contaminants should be proactively undertaken to determine potential for damages to multiple resources and loss of ecosystem services in sensitive environments.	
	• Species and habitats will become more valuable in light of GCC-induced shifts in biomes and predicted levels of the rates of extinction for certain species.	
	• Assessment and restoration and/or rehabilitation will need to incorporate the potential for wide variation in physical forcing factors such as temperature, contaminants, storms, and water quality/quantity in a changing landscape.	
	• The restoration and/or rehabilitation of ecosystem services in a changing landscape will require new and innovative approaches, adaptive management, and longer-term monitoring.	

### Occurrence, fate, and availability of chemicals

The effects of GCC on the occurrence, fate, and distribution of chemicals are anticipated to result in changes in exposures 21. These include the potential for increased global transport of dust and pollution 41, 42, increased erosion of soil and mobilization of legacy contaminants, alterations in the deposition and volatilization of chemicals, and altered flood and drought frequency and magnitude 5. Indirect effects have the potential to significantly modify the magnitude and temporal exposure to chemical contaminants. For instance, the potential for increased incidence of toxic algal blooms occurring more frequently and more severely in (1) warming, higher-latitude waters 43; (2) the influence of wind pattern–driven, upwelling processes leading to red tide or jellyfish-dominated offshore systems 44; (3) changes in pesticide-use patterns in response to changing agricultural practices and distributions of pests 40; and (4) modifications of food webs 45 leading to altered profiles of contaminant exposures. Moreover, GCC has allowed the opening of previously inaccessible resource-rich environments, which will inevitably lead to changing socioeconomic patterns of land use. Changes in the way that humans interact with environments will continue to lead to alterations in emissions of chemicals. For instance, as the ice-free season of the Arctic Ocean and the interior of Greenland increases 46, increased movement of ships through sensitive areas will increase the risk of spills or accidents. Iron and mercury sequestered in vegetation or detritus can be released by forest fires 47, while acidification of marine waters can release previously insoluble metals. The bioavailability of organic contaminants can be changed through alterations in pH or other biogeochemical processes, either increasing or decreasing depending on the chemical 48. Alterations in primary productivity in warming Arctic regions provide a matrix for the uptake of mercury and organochlorine chemicals and their concentration into food webs 49, 50. Changing trophic relationships within ecosystems may result in shifts in the dynamics of food webs and therefore influence the flow and accumulation of toxicants 45. Due to a variety of direct and indirect effects of GCC, it is anticipated that GCC will alter the distribution and bioavailability of chemical contaminants 51. Much of the data developed on GCC-related impacts to the fate and dynamics of chemicals can be used in current fate and transport models or with models that have been modified to account for GCC. However, a major gap exists with respect to the lack of atmospheric, soil, surface water, and other types of chemical monitoring data from developing countries; as a result, models for those regions of the globe remain underdeveloped 21.

#### Toxicology mechanisms

Effects of chemicals on an organism are a function of the mechanism of action and disposition of chemicals 52. The mechanism of action is the interaction of a chemical with its molecular target and the subsequent tissue, organ, and systemic perturbations that occur leading to a toxic response in an exposed organism 53. This interaction can be influenced by temperature (particularly in poikilotherms) and local tissue conditions such as level of hydration, lipid environment, and the general homeostatic condition of the organism 54, 55. Chemical disposition is a function of four factors: absorption, distribution, metabolism, and excretion in exposed organisms 56. The interaction of these four processes determines potential dose in the target tissue 57. Factors that modify the disposition of a chemical can increase uptake and retention or produce more reactive or toxic forms of the chemical. Dispositional factors are sensitive to temperature, water, and nutrient stresses that are anticipated with increased episodic occurrence of GCC-induced weather phenomena 58.

Both disposition and mechanism of action are influenced by a variety of factors such as water, food, temperature, competition, and predation. Physiological ecology, those biochemical and physiological processes that allow organisms to function within their ecological niche 59, may also be subject to the influence of GCC (e.g., changes in temperature) and from other environmental conditions that may be altered by GCC. These processes are key as their role in integrating internal homeostasis with the external environment represents a potentially important target for contaminants that is particularly sensitive to perturbations resulting from GCC 60.

Given the above discussion, biological responses to GCC and contaminants can be grouped into two major categories climate-induced toxicant sensitivity (CITS), where changes in climate may alter the ability of an organism to tolerate toxic insults, and toxicant-induced climate sensitivity (TICS), where exposure to toxic chemicals may alter the ability of an organism to tolerate the stressors associated with GCC 22. Integrating the concept of CITS and TICS with the recently developed framework for adverse outcome pathways could be an important step that would help to improve the understanding of GCC's potential influence on chemical contaminant toxicity as well as to demonstrate how contaminants might decrease the ability of an organism to withstand the effects of GCC 22. The adverse outcome pathway approach allows for both prospective assessments of potential interactions between GCC and chemicals and retrospective evaluations of observed findings believed to have a GCC etiology.

#### Populations and communities

A challenge for ecotoxicologists is to predict how joint effects of climatic stress and toxicants measured at the individual level (e.g., reduced survival or reproduction) will be transferred to the population (e.g., abundance, population growth rate, and extinction risk) and community (e.g., species richness, biodiversity, and food-web structure) levels 61. Increased temperature and other environmental impacts of GCC are also impacting processes in ecosystems and interactions between species (e.g., disrupting the timing of predator–prey interactions). Given the complexity and variability of impacts on the environment resulting from GCC, general predictions for interactions of GCC and toxicants at higher levels of biological scales may not be feasible at this time 23. Instead, it may be useful to consider different ecological mechanisms that are likely to influence responses to toxicants at the population and community levels under GCC. Stress due to altered climatic conditions may reduce the potential for tolerance to and recovery from exposure to toxicants. Long-term exposure to a toxicant may result in species being able to acquire tolerance to this stressor at the population or community level, but an associated “cost of tolerance” may be a reduced ability to tolerate subsequent climatic stress (or vice versa). Moreover, climate change induces large-scale shifts in the ranges of many species and thereby in community composition, which may affect the vulnerability of communities to other stressors. Ecological modeling based on species traits (representing life history, population vulnerability, sensitivity to toxicants, and sensitivity to climate change) can be a promising approach for predicting impacts of climate change and toxicants on populations and communities.

#### Human health risk assessment

In human health risk assessments, effects endpoints are typically more sensitive than mortality, reproductive disruption, and decreased growth, endpoints commonly assessed in nonhuman organisms. Human health effects are measured as increases in a wide variety of disease states and evaluated for the general population as well as highly susceptible subpopulations such as children, pregnant women, the elderly, and workers in high-exposure scenarios 62. Some exposure models incorporate details that mimic the lifestyles of the most sensitive individuals. Modeling of dose responses under conditions of GCC will, therefore, depend heavily on understanding how GCC modifies the exposure of humans to chemicals and other toxicants as well as the fate and transport of those chemicals or other toxicants in the human body. Unlike most organisms, humans have the ability to mitigate some, but not all, of their exposures to the stressors associated with GCC 24. As such, they represent a receptor that will challenge current approaches to characterizing exposure and effects in the conduct of human health risk assessment.

For both humans and nonhuman organisms, one possible scenario could arise where GCC-induced heat co-occurs with another stressor 34, 63 such as ozone, which can exacerbate the potential toxic effects of chemical contaminants. Poleward movement of disease vectors with a warming climate promises to increase the occurrence of vector-borne diseases into areas that previously had been free of them 64. Although weather and disease stressors are not new to public-health researchers, the frequency and range of occurrence will likely be increasing, demanding a better understanding of their effects on humans and sensitive subpopulations. These co-occurring stressors can affect the sensitivity of humans to chemicals and could be incorporated into the risk-characterization step of a human health risk assessment, evaluated in the uncertainty analysis or accounted for by using additional safety factors or other modifiers when establishing reference doses. With time and greater experience and understanding of the consequences of GCC to human health, epidemiologic studies could be designed to provide additional information on modeling parameters that could then account for major GCC effects in human health risk assessment 40.

#### Ecological risk assessment

In contrast to the singular human focus in human health risk assessments, an ecological risk assessment has a broader scope in its evaluation of risk, often integrating exposure and effect assessments over many species and biological scales, delineating criteria protective of varying proportions of species in the wild 27. The ecological risk-assessment analysis phase incorporates both exposure and effects assessments, making it amenable to using data that incorporate the effects of GCC in their development. Approaches to the assessment of risks from the effects of GCC, in the absence of chemicals, continue to develop and can provide inputs for contaminant-oriented assessments in the future 9, 14, 65. The ability to model the influence of GCC across exposure data is likely within grasp as much of the effort will focus on modifications of the bioavailability of contaminants. Alternatively, such modeling for adjusting effects endpoints will require a greater body of knowledge on how the variety of anticipated GCC stressors interact with contaminants in living organisms.

Assessments of ecological risks that are focused on GCC-induced stressors and endangered species, or those already documented to be adversely influenced by such stressors, are under development by a number of governmental agencies 4, 33, 66 and offer a logical mechanism by which assessments of contaminants might be incorporated. Depending on the focus of the ecological risk assessment, substantial effort may be required during the problem-formulation stage to ensure that all possible GCC stressor information, relevant to species or habitats of concern, is available for the assessment 25. It will also be important that future ecological risk assessments consider the risk-management scenario during the problem-formulation step to insure that the potential for GCC is included in the assessment.

#### Assessment of injury to natural resources and restoration ecology

Assessment of damage to and restoration of natural resources presents some of the more challenging situations, yet exciting opportunities, associated with the influence of GCC 26. Assessments of damage to natural resources share some characteristics with ecological risk assessments, particularly in the use of data on exposure and effects that can be influenced directly and/or indirectly by GCC 29, 67. Extensive data are used to characterize the spatial and temporal nature of damage to natural resources, incorporating past damage or harm since the initiation of contamination as well as projecting future losses of the resources until ecological restoration are complete. Differentiating the impacts of GCC on natural resources from those arising from exposure to chemical contamination is complicated but could be accomplished using historical data on anthropogenic stressors, where available, or in their absence estimating their types and magnitude that existed prior to the advent of GCC. Work will also be needed to understand the synergistic or antagonistic interactions between exposure to chemical contaminants and the effects of GCC (see previous discussion on CITS and TICS) and if the severity of chemical-induced damage may be influenced by differing GCC scenarios 68.

Ecological restoration and rehabilitation of previously contaminated sites will face challenges and opportunities in a future influenced by GCC. Shifting in the ranges of species and their assemblages (including migratory pathways and timing) resulting from changes in temperature and precipitation and the changes in habitat they engender, along with the ubiquity of some invasive species, will create increasingly difficult targets for restoration action 69. In some cases, it may become more difficult to find habitats suitable for restoration action, and some species may be forced into less desirable habitats as a result. Restoring ecosystem structure, function, and services may preclude the ability to completely restore assemblages of species that existed prior to the damage 70, 71. In developing diverse restored ecosystems with functional redundancy, it will be important to strive for the resilience necessary to buffer the dramatic climate changes predicted for the future 72. However, mitigating GCC and fostering adaptation to it can be achieved through the encouragement of restoration actions that optimize carbon sequestration (e.g., forest development on retired agricultural land) or provide corridors or expanded habitat for species with ranges stressed by GCC-altered temperature or hydrological patterns. Additional challenges will occur if GCC unfolds at different speeds and intensities around the globe, shifting baseline conditions and challenging assessments of damage to natural resources as well as how to restore those lost resources.

## CONCLUSION, UNCERTAINTIES, AND AREAS FOR RESEARCH

Human health and ecological risk assessments will become increasingly important in the anticipated development and evaluation of future actions that may be taken to support mitigation and adaptation to GCC. Such activities will require careful consideration of their repercussions (both positive and negative) and development of thorough assessments prior to and during implementation. For example, a risk assessment of GCC adaptation efforts in Bangladesh encouraging dike construction for flood control would need to consider how similar activities in that country have led to extensive leaching and contamination of groundwater with arsenic. Unfortunately, widespread arsenic poisoning in the country has resulted 73. Similarly, strategies to mitigate GCC that involve nano-iron seeding of ocean waters to stimulate algae growth and carbon sequestration may lead to extensive formation and release of neurotoxic domoic acid from diatom blooms 74. Such large-scale efforts to mitigate GCC are technically attractive but will undoubtedly test our ability to apply risk assessments and restoration strategies at landscape and global scales. Should GCC-induced tipping points be exceeded, it could lead to widespread or catastrophic environmental effects. At that point, risk assessors may be confronted with proposed mitigating or adaptive actions under emergency conditions with scant data to perform technically robust assessments.

Uncertainties remain in a number of factors that will need to be addressed in future decisions on research and policy. These include the rate at which GCC progresses as well as its spatial and temporal extent and variability, all of which will impact assessment of the fate and effects of chemicals as well as efforts on restoration and rehabilitation. The limited amount of GCC-relevant environmental data (e.g., monitoring of chemicals in the atmosphere, soils, and surface water) from developing areas of the world, where the impacts of GCC may be the most severe, increases uncertainty and represents a significant data gap that needs to be filled. The worldwide economic downturn at the time of this writing threatens funding at all levels 75 and, if not resolved, will lead to an ever-increasing number of data gaps, failures to improve monitoring and assessment techniques, and delays of restoration, rehabilitation, mitigation, adaptation, conservation, and protection projects that might otherwise alleviate or forestall the impacts of GCC. Funding for research and data collection that will lead to more targeted, cost-effective mitigation are issues that will challenge policy makers and governments around the globe.

Given the observed and predicted speed with which GCC is progressing 8, it is likely that many of the ideas developed now will be tested in the near future, leading to efforts to evaluate progress and update approaches and procedures in years to come. Future discussions and decisions dealing with important technical issues in environmental toxicology and chemistry would benefit from greater involvement of climate change scientists so that the potential for GCC to impact those technical issues is considered sooner rather than later. The reverse is true as well—it will become increasingly important for climate change scientists to involve environmental toxicologists, chemists, ecologists, and ecotoxicologists in their future assessments of impacts of GCC on humans and the earth's ecosystems.
